# Identification of the *Q* Gene Playing a Role in Spike Morphology Variation in Wheat Mutants and Its Regulatory Network

**DOI:** 10.3389/fpls.2021.807731

**Published:** 2022-01-11

**Authors:** Jiazi Zhang, Hongchun Xiong, Huijun Guo, Yuting Li, Xiaomei Xie, Yongdun Xie, Linshu Zhao, Jiayu Gu, Shirong Zhao, Yuping Ding, Luxiang Liu

**Affiliations:** National Key Facility for Crop Gene Resources and Genetic Improvement, National Center of Space Mutagenesis for Crop Improvement, Institute of Crop Sciences, Chinese Academy of Agricultural Sciences, Beijing, China

**Keywords:** wheat, mutant, *Q* gene, spike morphology, target genes

## Abstract

The wheat *AP2* family gene *Q* controls domestication traits, including spike morphology and threshability, which are critical for the widespread cultivation and yield improvement of wheat. Although many studies have investigated the molecular mechanisms of the *Q* gene, its direct target genes, especially those controlling spike morphology, are not clear, and its regulatory pathways are not well established. In this study, we conducted gene mapping of a wheat speltoid spike mutant and found that a new allele of the *Q* gene with protein truncation played a role in spike morphology variation in the mutant. Dynamic expression levels of the *Q* gene throughout the spike development process suggested that the transcript abundances of the mutant were decreased at the W6 and W7 scales compared to those of the WT. We identified several mutation sites on the *Q* gene and showed that mutations in different domains resulted in distinct phenotypes. In addition, we found that the *Q* gene produced three transcripts via alternative splicing and that they exhibited differential expression patterns in nodes, internodes, flag leaves, and spikes. Finally, we identified several target genes directly downstream of Q, including *TaGRF1-2D* and *TaMGD-6B*, and proposed a possible regulatory network. This study uncovered the target genes of Q, and the results can help to clarify the mechanism of wheat spike morphology and thereby improve wheat grain yield.

## Introduction

The yield of grass crops is influenced by several factors, and because inflorescences produce seeds and determine yield, modifying the morphological structure of inflorescences can directly improve seed yield (Wang and Li, 2008). Wheat (*Triticum aestivum* L.) is one of the most widely planted grain crops in the world, accounting for approximately 20% of all food calories consumed by humans (Braun et al., 2010). The yield of wheat is a complex trait determined by the number of spikes per unit area, number of grains per spike and 1,000 grain weight, and spike morphology can influence grain yield by affecting the number of spikelets, florets, and fertility (Xie et al., 2018; Cao et al., 2020). Therefore, it is important to modify the spike morphology and increase the number of spikelets to improve the yield.

Wheat domestication gene *Q* encodes an *APETALA2*-like transcription factor (TF) that controls domestication traits such as spike morphology, threshability, rachis fragility, plant height, and heading time, thereby affecting grain yield (Sears, 1954; Muramatsu, 1963; Kato et al., 1999, 2003; Faris and Gill, 2002; Faris et al., 2003, 2005; Xie et al., 2018; Debernardi et al., 2019; Liu et al., 2020). The *AP2* gene was first identified in *Arabidopsis* and is known to control flower and seed development (Jofuku et al., 1994). The *Q* gene has several *AP2* family homologs, including the *indeterminant spikelet1* (*ids1*) gene and *sister of ids1* (*sid1*) in maize as well as the *OsIDS* gene in rice (Chuck et al., 1998, 2008; Lee and An, 2012).

The domesticated *Q* allele was converted from the undomesticated *q* allele via amino acid mutations at position 329 and at the microRNA172 (miR172) target site (Simons et al., 2006; Sormacheva et al., 2015; Debernardi et al., 2017; Greenwood et al., 2017). The *Q* allele was shown to exhibit isoleucine at position 329, whereas valine resides at this position in the *q* allele (Simons et al., 2006). However, further study showed that this amino acid substitution may not affect the Q protein structure (Sormacheva et al., 2015). In addition, the *Q* allele was shown to have one more single nucleotide polymorphism (SNP) at the miR172 target site than the *q* allele (Sormacheva et al., 2015; Debernardi et al., 2017; Greenwood et al., 2017). This SNP in the *Q* allele was shown to induce an additional mismatch to miR172 and then reduce the cleaving efficiency, thereby increasing expression levels (Debernardi et al., 2017; Greenwood et al., 2017). Interestingly, the reduced miR172 activity resulted in a more compact spike and higher expression of *Q*, indicating that the miRNA plays a crucial role in wheat domestication (Debernardi et al., 2017; Greenwood et al., 2017).

In addition to *Q* and *q*, there are several alleles of the *Q* gene. Greenwood et al. (2017) found a dwarf, compact spike mutant in a sodium azide-induced M_2_ population, which had an SNP at the miR172 target site of the *Q* gene. This SNP reduced miRNA-dependent degradation and increased the protein abundance of *Q* and was named the *Q’* allele. Jiang et al. (2019) identified a new allele, *Q^t^*, in Tibetan semi-wild wheat that had a 161-bp transposon insertion in exon 5 and had no functional ability.

Recently, several studies have focused on the interaction network of the *Q* gene. MiR172, upstream of the *Q* gene, can downregulate the expression of *Q* directly by miRNA-dependent degradation (Debernardi et al., 2017; Greenwood et al., 2017; Liu et al., 2018). A transcriptome study found that the levels of epigenetic-associated genes were upregulated while those of photosynthesis- and cell wall-associated genes were downregulated in the *q* allele (Zhang et al., 2020). In addition, many TFs are differentially expressed between the *Q* and *q* alleles, such as those of the MYB and basic helix–loop–helix (bHLH) classes (Zhang et al., 2020). Recently performed yeast two-hybrid assays identified a bHLH family TF, TaLAX1, that can interact with the Q protein to oppositely regulate wheat domestication traits. Moreover, TaLAX1 and Q can antagonistically regulate the transcription of the lignin biosynthesis-related genes *TaKNAT7* and *TaPAL1*, which were shown to be differentially expressed in previous transcriptome data (He et al., 2021).

Here, we identified a new allele of the *Q* gene in a wheat speltoid spike mutant using bulked segregant analysis (BSA) and gene mapping. To compare the phenotypic variation and transcript levels of *Q* between the WT and mutant, we used qRT-PCR to quantify the dynamic expression levels during spike development. In addition, we identified a series of *Q* alleles in the mutant population and then used them in combination with phenotypic data to analyze the functions of different sites in the *Q* gene. Finally, DNA affinity purification sequencing (DAP-seq) was performed, revealing several downstream target genes, including *TaGRF1-2D* and *TaMGD-6B*. This study identified downstream target genes of Q and improved our understanding of the mechanism underlying spike morphology and grain yield potential.

## Materials and Methods

### Plant Materials and Phenotypic Analysis

The spike morphology mutant *je0275* was obtained by mutagenesis of the winter bread wheat cultivar Jing 411 (J411) with ethyl methane sulfonate (EMS). The EMS treatment was performed following our previous method (Xiong et al., 2018). In brief, J411 wheat seeds were immersed in water for 16 h and then were soaked in 1.5% EMS (Sigma, United States) for 4–8 h. The treated seeds were washed for 5 min with running water and then planted to well-managed field with several generations. For gene mapping, the *je0275* mutant was reciprocally crossed with J411 to produce two F_2_ populations. These populations (642 plants and 200 plants) and the parent lines were sown at the Zhongpuchang station of the Institute of Crop Sciences, Chinese Academy of Agricultural Sciences (Beijing, China) and grown under well-managed field conditions. Each line was planted with 20 plants in a row of 2 m. Spike morphologies were observed at the spike filling stage in both 2018 and 2020.

### Bulked Segregant Analysis

Genomic DNA was extracted from the flag leaves of each plant at the heading stage using the PVP40 method as previously described (Li et al., 2017), and the extracted DNA samples were used for construction of bulks. Based on spike morphology, four bulks from two F_2_ populations were designed: two wild-type bulks (each containing 50 normal square-headed plants) and two mutational bulks (each containing 50 plants that had a speltoid-like spike phenotype). These four bulks were constructed by mixing 500 ng of DNA from each selected plant. A total of four bulks and 2 parent lines were subjected to whole-exome sequencing on the Illumina HiSeq X platform. Approximately 14.03 Gb of clean reads were obtained by removing low-quality reads and reads with adapters. All of the clean reads were aligned to the Chinese Spring v1.1 genome released by the International Wheat Genome Sequencing Consortium (IWGSC)^1^ using Burrows-Wheeler Aligner (BWA) software. The alignment data were converted to BAM files using SAMtools, and repetitive reads were excluded using Biobambam2. In total, 1,795,250 SNPs were obtained from all four bulks and two parent lines. We used two standards to filter low-quality or low-confidence data: sequencing depth and genotypes of the two parent lines. A total of 1,604,480 SNPs were filtered out due to having a sequencing depth < 4 in the parent lines. In addition, 126,574 loci with the same genotype between the two parent lines were discarded. Finally, 64,196 SNPs with high quality and high confidence distributed on all 21 chromosomes were selected for further analysis. The genotype frequency between two progeny bulks with different spike phenotypes was calculated by the ΔSNP-index. To eliminate the effects of false positive loci, locally weighted scatterplot smoothing (LOESS) fitting of the absolute value of the ΔSNP-index was used to calculate the candidate positions.

### Phenotype Identification of Ethyl Methane Sulfonate-Induced Mutated Populations and Target Gene Sequencing

Our laboratory built a M_7_ mutated population by using EMS to induce J411, and this population was genetically stable due to the high generation. We selected 41 lines based on spike morphology variation, including 27 compact spike lines and 14 speltoid spike lines. Genome-specific primer pairs were used to amplify the full-length sequences of the *5AQ* and *5Dq* genes (Supplementary Table 1). Sanger sequencing was performed with two replicates to discover mutations between J411 and the selected lines. Then, the mutations were combined with phenotype variations to analyze the effects of the mutations.

### Kompetitive Allele Specific PCR Assays

Based on the whole-exome sequencing data, SNPs between two parent lines located on the candidate chromosome were selected for conversion to Kompetitive Allele Specific PCR (KASP) markers using the PolyMarker web interface.^2^ Then, the FAM or HEX tail was added to the 5′ end of two forward KASP primers (FAM tail: 5′ GAAGGTGACCAAGTTCATGCT 3′; HEX tail: 5′ GAAGGTCGGAGTCAACGGATT 3′). Two parent lines were used to screen specific KASP primers (Supplementary Table 1). The CFX 96 Real-Time System (Bio Rad, United States) was used to perform PCR assays and fluorescence detection. The KASP reaction system contained 2.5 μL KASP master mix (LGC Genomic, United Kingdom), 0.06 μL primer mix [primer A (10 μM): primer B (10 μM): primer R (10 μM) = 12: 12: 30], 0.04 μL MgCl_2_ (50 mM) and 2.4 μL genomic DNA (60 ng⋅μL^–1^). PCR were performed as described previously (Li et al., 2020).

### RNA Isolation

Young spikes of J411 and *je0275* were sampled at Waddington scales W3, W3.5, W4, W5, W6, W7, W9, W9.5, and W10 (Waddington et al., 1983). Because individual samples were too small for direct RNA extraction, we sampled more than 70 young spikes from the W3 and W3.5 scales and over 40 spikes from the W4 scale. These samples were then divided into two biological replicates. At the W5 scale, we mixed over 25 spikes and divided them into three biological replicates. At the W6, W7, W9, and W9.5 scales, four spikes were mixed together as one biological replicate, and three biological replicates were sampled. Flag leaves, the first internodes and the second nodes of J411 were sampled at heading stage. Total RNA was extracted from each sample using the RNeasy Plant Mini Kit (Qiagen, Germany).

### Reverse Transcription and Quantitative Real-Time PCR Analyses

We used the PrimeScript RT reagent Kit with gDNA Eraser (TaKaRa, China) to remove gDNA and synthesize first-strand cDNA. Quantitative real-time PCR was conducted with PerfectStart Green qPCR SuperMix (TransGen Biotech, China) on the CFX 96 Real-Time System (Bio Rad, United States). Reverse transcription and qPCR were performed according to the manufacturers’ protocols. In this experiment, ACTIN was used as endogenous control gene for expression normalization and at least three technical replicates were performed for each sample (Supplementary Table 1). Relative expression levels were calculated using 2^–ΔΔ*CT*^ method (Livak and Schmittgen, 2001).

### RNA-Seq and Data Analysis

A total of six RNA samples, including three biological replicates of WT and *je0275* at the W6 scale, were used for RNA-seq, which was performed on an Illumina HiSeq platform using the paired-end 150 bp (PE150) strategy. Approximately 66 Gb of raw reads were obtained from the six samples. After excluding low-quality reads, 60.81 Gb of clean reads were obtained from the six samples, and each sample had approximately 10 Gb of clean reads. The Chinese Spring reference genome sequence v1.1 was obtained from the IWGSC. All of the clean reads from the six samples were aligned to the reference genome using the HISAT2 program (Kim et al., 2019). The fragments per kilobase of exon model per million mapped fragments (FPKM) value of each of the three biological replicates was calculated to estimate gene expression levels (Trapnell et al., 2010). The *p*-values were adjusted for multiple comparisons using the false discovery rate (FDR). Differentially expressed genes (DEGs) were indicated by their expression levels being changed by twofold and their FDR being less than 0.05.

### DNA Affinity Purification Sequencing and Data Analysis

#### Library Preparation

Genomic DNA was extracted from the young spikes of J411 using the cetyltrimethylammonium bromide (CTAB) method (Saghai-Maroof, 1985) and sheared to 300–500 bp fragments using BioRuptor Plus (Diagenode, United States). The fragment size was selected using Mich NGS clean beads (Mich, China). We used the NEXTflex Rapid DNA-Seq Kit (PerkinElmer, Inc., United States) to repair fragment ends and then used NEXTflex ligase Enzyme Mix (PerkinElmer, United States) to add the adapter and construct the library.

#### Protein Expression and Binding

The full-length coding sequence (CDS) of the *Q* gene was cloned from the cDNA of J411 and confirmed by Sanger sequencing. Then, the CDS of *Q* was ligated into the pFN19K HaloTag T7 SP6 Flexi expression vector (Promega, United States) using the ClonExpress MultiS One Step Cloning Kit (Vazyme, China). The Halo-Q fusion protein was expressed using the TnT SP6 High-Yield Wheat Germ Protein Expression System (Promega, United States) in a 50 μL expression system that included 2,000 ng of vector and 30 μL of expression buffer by incubating at 37°C for 2.5 h. Then, Magne HaloTag Beads (10 μL, Promega, United States) were directly added to the expression reaction and incubated for 1 h at 25°C to capture the Halo-Q fusion protein. After washing three times with equilibration buffer (Mich, China), 25 μL of the DNA library was added and incubated for 1 h at 25°C. Then, the beads were washed three times with equilibration buffer (Mich, China) to remove non-specifically bound DNA fragments. The binding fragments were released by heating to 98°C for 10 min and then transferred to a new tube. To enrich the fragments and add the index, PCRs were performed using the KAPA HiFi HotStart ReadyMix PCR Kit (Roche, Switzerland). Finally, the binding fragments were sequenced on an Illumina NavoSeq platform using the PE150 strategy. The original genomic DNA library without protein binding was sequenced as the negative control.

#### Sequencing Data Analysis

A total of 10.48 Gb of clean reads were obtained and mapped to the CS v1.1 reference genome using BWA-MEM (Vasimuddin et al., 2019). Peak calling was performed using Macs2 (Zhang Y. et al., 2008). We located peaks to the gene model and calculated the distance to the transcription start site (TSS) using Homer (Heinz et al., 2010). Motif calling was performed using Meme-Chip software (Machanick and Bailey, 2011).

## Results

### Gene Mapping and Identification of the *Q* Gene Controlling Spike Morphology Variation in a Wheat Mutant

**FIGURE 1:**
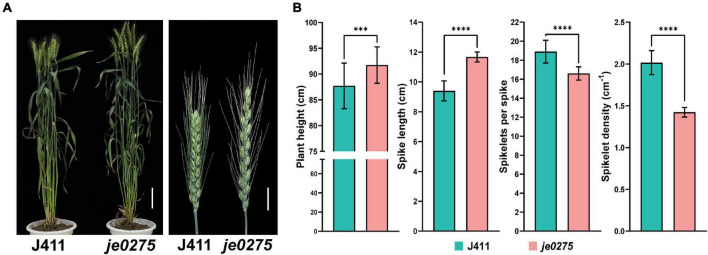
Phenotypic analyses of the wheat lines used in this study. **(A)** Phenotype of the speltoid spike mutant *je0275* at the heading stage. Bars, 10 cm (left) and 2 cm (right). **(B)** The plant heights, spike lengths, numbers of fertile spikelets per spike and spikelet densities of *je0275* and WT plants, grown in approximately 10 rows, were measured. The data are presented as the mean, and the error bars indicate the SD. *** and **** indicates significant differences at the 0.001 and 0.0001 level (Student’s *t-*test), respectively.

We identified a wheat mutant, *je0275*, showing speltoid spike morphology resulting from EMS mutagenesis (Figure 1A). In contrast to those of the wild-type (WT) J411 plant, the plant height and spike length of *je0275* were significantly increased, but its number of fertile spikelets per spike and spikelet density were significantly decreased (Figure 1B). The 1,000 grain weight values did not significantly differ between *je0275* and J411 (Supplementary Figure 1). To map the gene controlling spike morphology in *je0275*, two F_2_ populations with 642 plants and 200 single plants were generated by the reciprocal crossing of *je0275* and J411. Two kinds of spike morphology segregated in the reciprocally crossed F_2_ populations, and the ratio of speltoid spikes to normal spikes was 3–1, as verified by the chi-square test (Supplementary Table 2). This ratio indicated that this spike morphology variation was controlled by a single gene and that the speltoid spike phenotype resulted from a dominant allele.

**FIGURE 2:**
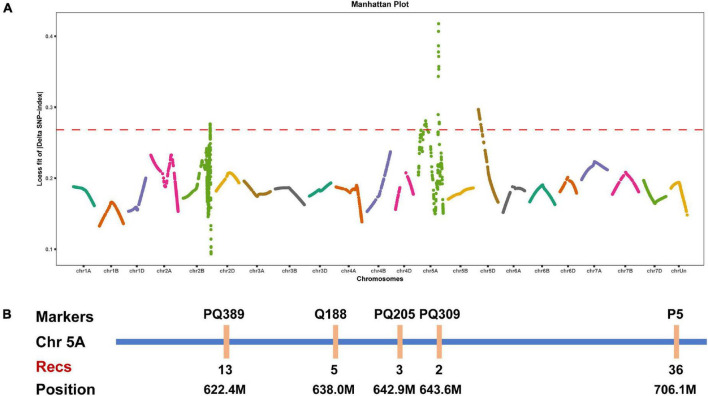
Elucidation of the gene controlling speltoid spikes via BSA and linkage mapping. **(A)** Distribution of the LOESS fits of the ΔSNP-index values on all chromosomes. The names of all chromosomes are shown on the *x*-axis, and the LOESS fits of the ΔSNP-index values are shown on the *y*-axis. **(B)** Linkage map around the candidate interval. Recs indicates the recombinants.

BSA is a rapid strategy to map genes of interest based on comparisons with traditional genetic linkage mapping (Giovannoni et al., 1991; Michelmore et al., 1991; Wenger et al., 2010; Trick et al., 2012). We herein performed whole-exome sequencing for BSA to map the gene controlling the speltoid spike phenotype in *je0275*. Fifty normal spike plants and 50 speltoid spike plants from each reciprocal crossed F_2_ population were selected for the construction of two bulks. A total of four progeny bulks and two parent lines were used for whole-exome sequencing. The LOESS fit result showed that the end of chromosome 5A had a significant peak associated with spike morphology variation (Figure 2A).

We developed several molecular markers on the long arm of chromosome 5A to verify the BSA mapping result. Polymorphic SNPs between two parent lines were selected for conversion to KASP markers using the PolyMarker website and then tested by the parents. Finally, five specific KASP markers were obtained and then genotyped in the F_2_ population. According to the phenotype and genotype of each marker, the lowest number of recombinants was observed in marker PQ309, indicating that the candidate gene was located between markers PQ205 and P5, corresponding to positions 642.9–706.1 Mb on chromosome 5A (Figure 2B). Notably, the wheat domesticated gene *Q* was located in this region, and the phenotype of the undomesticated *q* allele was very similar to that of *je0275*. We speculated that the *Q* gene was the candidate gene affecting spike morphology variation in *je0275*.

**FIGURE 3:**
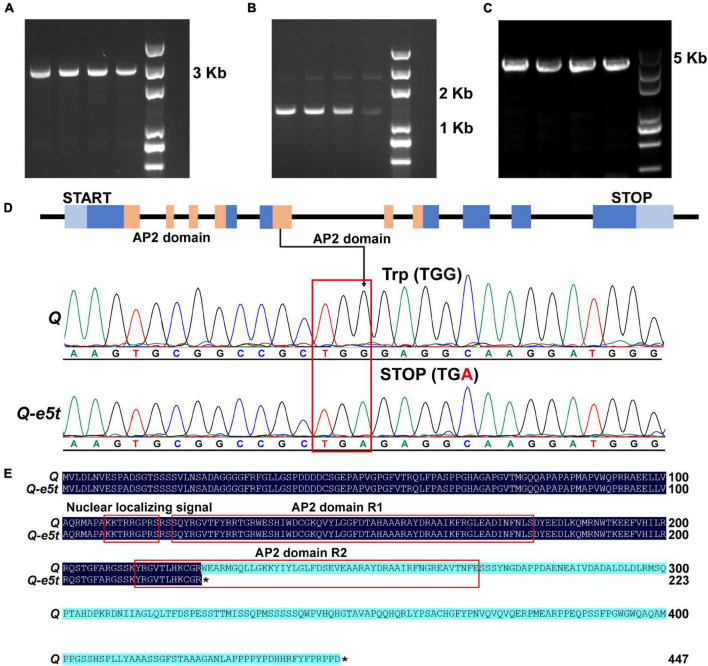
Amplification of the *Q* gene and mutation detection. **(A,B)** Primers used to amplify the full-length sequence of the *5AQ* gene. **(C)** Primers used to amplify the full-length sequence of the *5Dq* gene. **(D)** The G-to-A mutation existed in the *Q-e5t* allele. **(E)** Protein sequence alignment of the *Q* and *Q-e5t* alleles. * indicates the stop codon of each peptide.

To determine whether the *Q* gene was mutated, two pairs of specific primers were designed to amplify the full-length sequences of the *Q* gene in *je0275* and J411 (Figures 3A,B and Supplementary Table 1). In addition, to verify whether mutations existed in *5Dq* homologs, one pair of specific primers developed in a previous study was used for amplification (Figure 3C and Supplementary Table 1). The Sanger sequencing results revealed a G-to-A mutation in exon 5 of the *5AQ* gene in *je0275*; this mutation resulted in translation termination and was named *Q-e5t* (Figure 3D). In contrast to the full-length 447 amino acids in the WT, the Q protein of *je0275* was truncated to 223 amino acids and was missing one *AP2* domain (Figure 3E). We speculated that the phenotypic variation in *je0275* was derived from the mutation resulting in a stop codon in exon 5 of the *5AQ* gene.

**FIGURE 4:**
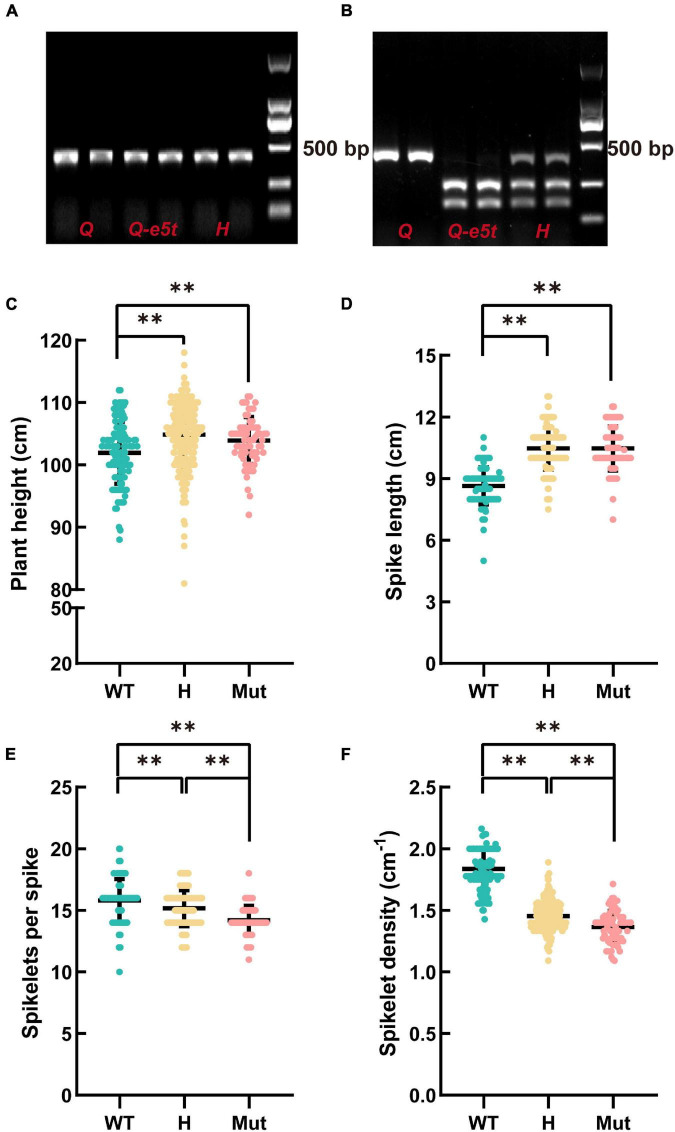
Development of the diagnostic marker Q-CAPS-3 and the phenotypic characteristics of the F_2_ population. **(A)** The PCR product of Q-CAPS-3. **(B)** The cleavage product of the *Dde*I restriction enzyme. H indicate the *heterozygous* genotype. **(C–F)** The plant heights, spike lengths, numbers of fertile spikelets per spike and spikelet densities of different genotypes. WT, Mut and H indicate the *Q* allele, *Q-e5t* allele and *heterozygous* genotype, respectively. ** indicates significant differences at the 0.01 level (Student’s *t*-test).

The mutation in *5AQ* produced a cutting site for the *Dde*I restriction enzyme, while the WT sequence could not be cut by *Dde*I. Therefore, we developed a specific cleaved amplified polymorphic sequence (CAPS) marker to identify *Q* and *Q-e5t* alleles. We amplified 395 bp PCR products and then used the *Dde*I restriction enzyme for cleavage (Figure 4A). The *Q* allele had only one band at 395 bp, while the *Q-e5t* allele exhibited two bands at 258 and 140 bp, and the heterozygous genotype included three bands after digestion by the restriction enzyme (Figure 4B). We then identified the genotypes of the F_2_ population and found that spike morphology was completely linked with the genotypes of the *Q* gene (Supplementary Table 3). Taken together, these results led to the conclusion that mutation of the *5AQ* gene resulted in spike morphology variation in *je0275*.

### Analyzing the Pleiotropic Effects of the *5AQ* Gene

The *Q* gene encodes an AP2 family TF that controls many agronomic traits, such as plant height, threshability and glume tenacity (Muramatsu, 1963; Kato et al., 1999, 2003; Faris and Gill, 2002; Faris et al., 2003, 2005; Xie et al., 2018; Liu et al., 2020). To confirm whether the mutation in the *Q* gene influences plant height, spike length, number of spikelets per spike, and spikelet density, we investigated these four traits of 403 plants in the F_2_ population. The statistical results showed that the average plant height and spike length of the *Q-e5t* allele were significantly higher than those of the *Q* allele. Conversely, the average number of spikelets per spike and spikelet density of the *Q-e5t* allele were significantly lower than those of the *Q* allele (Figures 4C–F). These results indicated that the mutation in the *Q* gene also affected the plant height, spike length, number of spikelets per spike, and spikelet density.

### Identification of *Q* Gene Mutations in the Wheat Mutant Population

**FIGURE 5:**
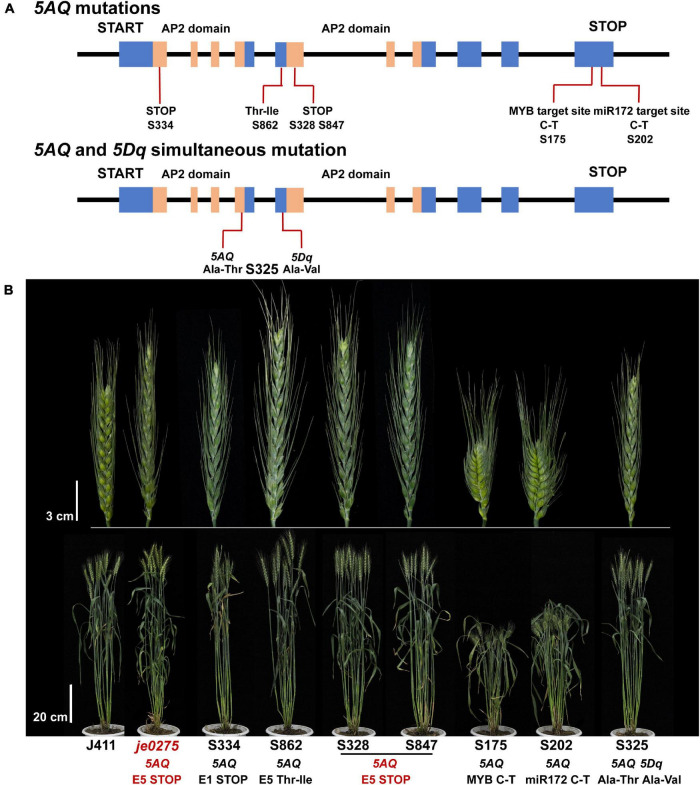
Identification of mutations in the *Q* gene. **(A)** The mutation sites and predicted translational changes in mutants are marked in the schematic representation of the *Q* gene. The blue rectangles indicate exons, the orange rectangles indicate the *AP2* domains, and the black lines indicate introns of the *Q* gene. **(B)** Phenotype of mutants that had mutations in the *Q* gene at the heading stage. Bars, 3 cm (upper) and 20 cm (lower).

Mutated populations are an essential tool of functional genomic research and a crucial resource for crop breeding and genetic improvement (Guo et al., 2017, 2021; Xiong et al., 2018). Selecting mutants with phenotypes related to the *Q* gene in mutated populations and then screening for *Q* gene mutations are important for discovering other alleles of the *Q* gene and verifying the candidate gene. We selected 41 lines showing spike morphology variation from a wheat mutant population to analyze mutations of the *Q* gene. In total, 10 lines had mutations in the *5AQ* gene, and one line had simultaneous mutations in both the *5AQ* and *5Dq* genes (Figure 5 and Supplementary Table 4). In the 10 mutant lines with *5AQ* mutations, 8 had mutations in two *AP2* domains or their connected region showing speltoid spikes. Lines S316, S328, S847, S850, S856, and S859 had the same mutation and phenotype as *je0275*. S202 had a mutation at the miR172 target site in exon 10 and showed a compact spike, consistent with a previous study (Debernardi et al., 2017; Greenwood et al., 2017; Liu et al., 2018). In addition, S175 had a mutation at the predicted MYB target site in exon 10 and showed a compact spike. S325 had simultaneous mutations in both *5AQ* and *5Dq* genes and showed a more compact spike and lower plant height than the single mutation in *5AQ*.

Moreover, we identified four mutant lines with mutations in *5AQ* in an M_2_ population. All four lines had missense mutations in *AP2* domains and showed a speltoid spike phenotype. Interestingly, the line D15941 had a heterozygous genotype in the M_2_ plant, and its M_3_ family segregated the speltoid spike and normal spike phenotypes (Supplementary Figure 2).

### Dynamic Expression Levels During Spike Development

**FIGURE 6:**
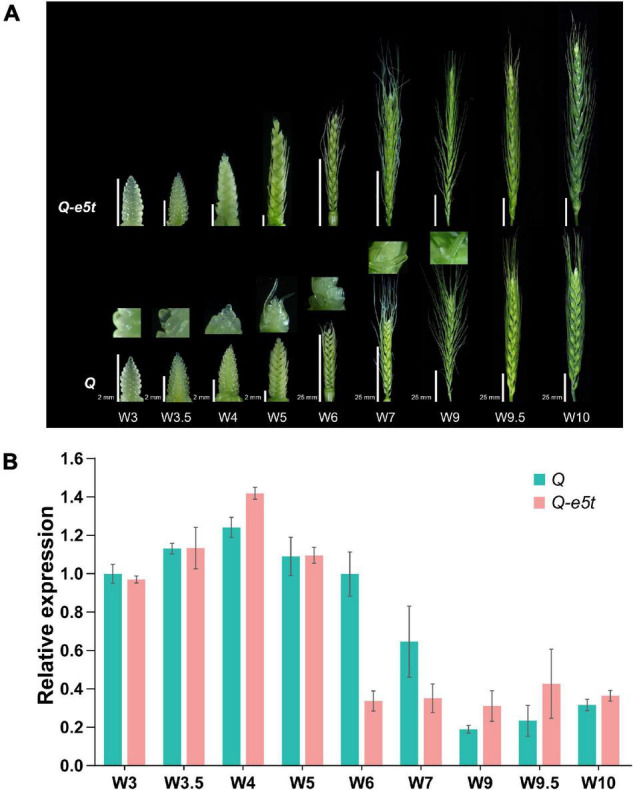
Phenotypes and relative expression levels during young spike development. **(A)** The phenotypes of young spikes at the Waddington scale W3, W3.5, W4, W5, W6, W7, W9, W9.5, and W10. Bars, 2 mm (W3, W3.5, W4, and W5) and 25 mm (W6, W7, W9, W9.5, and W10). *je0275* and WT exhibited the same bars at the same scale. **(B)** The relative expression levels of the *Q* and *Q-e5t* alleles from stages W3 to W10 were measured by qRT-PCR. The data are presented as the mean, and the error bars indicate the SD.

To identify the expression levels of the *Q* and *Q-e5t* alleles during spike development, we extracted young spikes from the glume primordia stage (W3) to the pollination stage (W10) and compared their spike morphologies at the different development stages (Figure 6; Waddington et al., 1983). The dynamic expression levels of the *5AQ* gene were quantified by real-time qPCR. The expression level of the *Q* allele was increased from the W3 to W4 scales, decreased from the W5 to W7 scales, and then maintained a low level at the W9 and W10 scales, which was consistent with the expression pattern in the Chinese Spring genome (Figure 6B; Simons et al., 2006). The transcription levels of *Q-e5t* were the same as those of the *Q* allele from the W3 to W5 scales but were reduced to the low level earlier at the W6 scale and maintained this low level from the W6 scale to the W10 scale (Figure 6B). Comparing the spike morphologies of *je0275* and WT from the W3 to W10 scales showed that the speltoid phenotype occurred at the W5 scale and was completely formed at the W6 scale (Figure 6A).

### Alternative Splicing Patterns of the *Q* Gene

**FIGURE 7:**
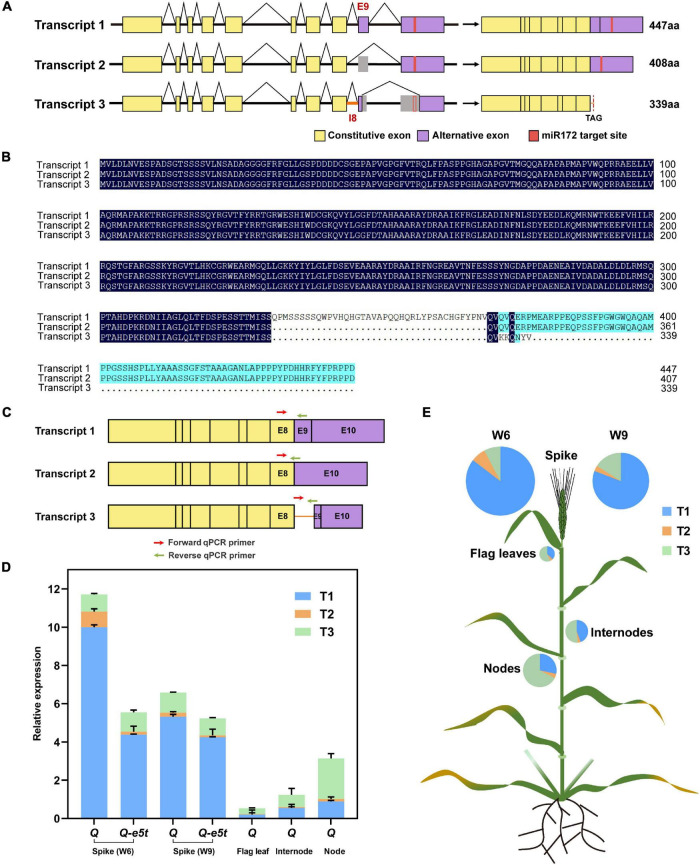
The alternative splicing patterns of the *Q* gene. **(A)** The *Q* gene produced three transcripts, T1, T2, and T3, through alternative splicing. The yellow rectangles indicate constitutive exons, the purple rectangles indicate alternative exons and the red rectangles indicate the miR172 target site of the *Q* gene. **(B)** Protein sequence alignment of the three transcripts. **(C)** Three pairs of specific qPCR primers are indicated on the schematics of three transcripts. The red arrows indicate the forward qPCR primers, and the green arrows indicate the reverse qPCR primers. **(D)** The relative expression levels of the three transcripts of the *Q* and *Q-e5t* alleles in spikes (W6 and W9 scales), flag leaves, internodes and nodes. The data are presented as the mean, and the error bars indicate the SD. **(E)** The expression levels of the three transcripts are indicated in the wheat schematic. The sizes of the pies indicate the total expression levels of all the transcripts.

The *Q* gene was shown to generate three transcripts by alternative splicing as determined by RT-PCR (Supplementary Figure 3). The first transcript (T1) was the full-length coding sequence containing 10 exons and encoded the 447 amino acid protein. The second transcript (T2) skipped exon 9 and encoded a 408 amino acid protein. The third transcript (T3) retained intron 8, skipped half of exons 9 and 10 and lost the miR172 target site, but a stop codon in intron 8 resulted in early termination at amino acid position 340 (Figures 7A,B). To investigate the expression levels of the three transcripts at different stages and tissues, we developed two other pairs of specific qPCR primers for transcripts 2 and 3 at their splicing regions (Figure 7C and Supplementary Table 1). We first compared the transcript levels of all three transcripts between the *Q-e5t* and *Q* alleles at the W6 and W9 scales of spike development. The full-length CDS (T1) had the highest expression level in both spike stages. T2 had the lowest expression level and showed the same trend as T1. The expression levels of T3 were the same at the two stages (Figure 7D). We found that *Q* and *Q-e5t* showed similar alternative splicing patterns (Figure 7D). Then, we identified the expression levels in different tissues, revealing that the total expression levels in flag leaves, internodes and nodes were lower than those in spikes. In contrast, T3 was expressed at the highest levels in the other three tissues, and T1 expression was lower than that of T3 (Figures 7D,E). Therefore, we concluded that the mutation in the *Q-e5t* allele did not influence the alternative splicing of the *Q* gene, and tissues other than spikes, such as flag leaves, internodes and nodes, had different alternative splicing patterns.

### Genome-Wide Identification of the Direct Downstream Targets of Q

**FIGURE 8:**
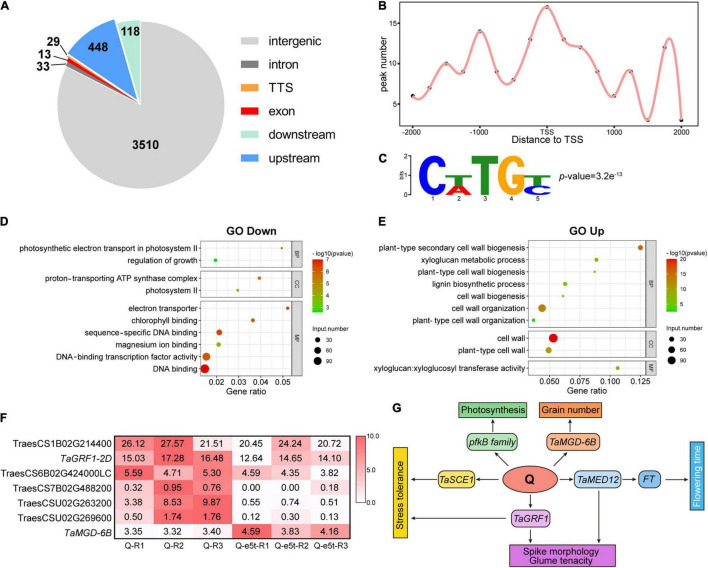
Identification of genome-wide downstream targets of Q. **(A)** Genome-wide distribution of Q binding peaks. **(B)** The binding performance of Q in gene models. The *x*-axis indicates the distance to the TSS, and the *y*-axis indicates the number of peaks. **(C)** Q binding to the CTTGC core motif. The *p*-value was calculated by MEME. **(D,E)** GO enrichment analyses of down- and upregulated genes. The bubble size indicates the number of differentially expressed genes. The gene ratio is shown on the *x*-axis, and BP, CC, and MF on the *y*-axis indicate the biological process, cellular component and molecular function categories, respectively. **(F)** Heatmap showing the expression of 7 target genes bound by Q. The colors in the heatmap indicate the FPKM value. **(G)** Possible regulatory network of the *Q* gene.

While the *Q* gene has been reported to control domestication traits such as spike morphology and threshability, the target genes and underlying mechanisms remain unclear. To investigate the regulatory mechanisms underlying domestication traits, we performed DAP-seq to identify genome-wide targets of Q. A total of 4,151 peaks downstream of Q were identified on 21 chromosomes (Supplementary Dataset 1). We used the distance to the TSS to analyze the genomic distribution of the peaks, revealing 630 peaks that were located in genic regions 10 kb upstream to 5 kb downstream of the TSS, including exons, introns and transcription termination sites (Figure 8A and Supplementary Dataset 2). Among the peaks located in the proximal promoter region (2 kb upstream), Q showed a strong binding preference to the TSS and 1 kb upstream of the TSS (Figure 8B). Based on this binding preference, we defined putative target genes as their corresponding peaks mapping from 2 kb upstream to 500 bp downstream of the TSS. In total, 112 genes were identified as putative target genes controlled by Q (Supplementary Dataset 3). To predict the binding site of Q, we used Meme-Chip software (Machanick and Bailey, 2011) to analyze the genome-wide sequence bound by Q, and CTTGC was identified as the most enriched binding motif (Figure 8C).

To further investigate the regulatory models of the putative Q target genes, RNA-seq analyses of young WT and *je0275* spikes were performed at the W6 scale (Supplementary Dataset 4). Gene Ontology (GO) enrichment analysis showed that the downregulated DEGs in *je0275* comparing to WT were related to TF activity and the regulation of growth and photosynthesis, while the upregulated DEGs were related to the cell wall (Figures 8D,E). Among the 112 putative target genes identified by DAP-seq, seven had significant differences in expression levels, including six that were downregulated and one that was upregulated in *je0275* (Figure 8F). The six downregulated target genes were TraesCS1B02G214400, TraesCS2D02G246600, TraesCS6B02G424000LC, TraesCS7B02 G488200, TraesCSU02G263200, and TraesCSU02G269600. TraesCS1B02G214400 was annotated as a SUMO-conjugating enzyme in *Aegilops tauschii* that is involved in responses to drought stress in rice (Nurdiani et al., 2018). TraesCS6B02G424000LC was annotated as a putative En/Spm subclass transposon protein. TraesCS7B02G488200 was annotated as a pfkB-like carbohydrate kinase family protein that is involved in plant growth and chloroplast development in *Arabidopsis* (Arsova et al., 2010). TraesCSU02G263200 was annotated as a subunit 12 of mediator of RNA polymerase II, which enhances the *FT* regulating flowering time in *Arabidopsis* (Imura et al., 2012). TraesCSU02G269600 was annotated as 3-oxoacyl-[acyl-carrier-protein] synthase 1. Notably, we found that *TaGRF1-2D* (TraesCS2D02G246600) was downregulated in *je0275* (Huang et al., 2021). *TaGRF1-2D* is a close homolog of *OsGRF10*, which is involved in floral organ development in rice (Liu et al., 2014). In addition, we found that the monogalactosyl diacylglycerol (MGD) synthase *TaMGD-6B* (TraesCS6B02G404700), which controls the number of grains per spike and grain weight per plant in wheat, was bound by Q and upregulated in *je0275* (Du et al., 2020). Thus, we concluded that the *Q* gene regulates a variety of traits, including spike morphology, threshability, heading time, and grain yield, by interacting with these genes.

## Discussion

### The *Q* Gene Was Shown to Be Responsible for the Spike Morphology Variation in *je0275*

Wheat yield is attributed to the number of spikes per unit area, the number of grains per spike and the 1,000 grain weight, but increasing yield by increasing the grain weight is difficult (Frederick and Bauer, 1999; Fischer, 2011). Spike morphology can influence the number of grains per spike and thus the yield by affecting traits such as the spike length and spikelet number (Xie et al., 2018). Here, we identified the EMS-induced wheat mutant *je0275*, which exhibited a speltoid spike morphology (Figure 1A). The *je0275* mutant exhibited a significantly increased plant height and spike length, while the fertile spikelets per spike and spikelet density were significantly decreased (Figure 1B). To map the gene controlling spike morphology variation in *je0275*, we performed whole-exome sequencing and BSA of two reciprocally crossed F_2_ populations. By combining BSA and genetic mapping, the candidate gene was finally located within the 642.9–706.1 Mb interval on chromosome 5A, which included the domesticated gene *Q* (Figures 2A,B). The undomesticated *q* allele was characterized by a speltoid spike morphology and non-free-threshing, which was very similar to *je0275*. Further Sanger sequencing revealed a G-to-A mutation in exon 5 of the *Q* gene in *je0275*, which resulted in translation termination (Figures 3D,E). Diagnostic markers were developed based on this mutation, and its genotype was completely associated with spike morphology in the F_2_ population, indicating that the *Q* gene controlled the spike morphology variation in *je0275* (Figures 4A,B and Supplementary Table 3).

To further determine whether the *Q* gene controls the plant height, spike length, number of fertile spikelets per spike, and spikelet density variation, we measured these traits in an F_2_ population and considered the genotype of the *Q* gene to verify their linkage. The results showed that these traits were linked to the genotype of the *Q* gene, and the trend was the same as that of the parents (Figures 4C–F). A previous study analyzed the agronomic traits of the *Q* gene using a recombinant inbred line population and found that the *Q* allele had a higher spike compactness and 1,000 grain weight, while the *q* allele had a higher spike length and number of grains per spikelet/spike (Xie et al., 2018). Herein, the 1,000 grain weights did not significantly differ between WT and *je0275*, probably due to different mutation sites or genetic backgrounds.

### Different Allele Variations in the *Q* Gene Resulted in Distinct Phenotypes

In the 10 mutant lines with *5AQ* mutations, 8 had mutations in two *AP2* domains or their connected region, and all showed similar speltoid spikes (Figure 5 and Supplementary Table 4). AP2 family TFs are distinguished by two conserved *APETALA2* (*AP2*) DNA-binding domains with critical and indispensable functions (Jofuku et al., 1994; Riechmann and Meyerowitz, 1998). The lines S334, S316, S328, S847, S850, S856, and S859 had mutations in *AP2* domains, and all showed speltoid spikes. Notably, several lines had the same mutation and phenotype as *je0275*, confirming that the speltoid spike of *je0275* was caused by this mutation. The region connecting the two *AP2* domains, called a linker, is also conserved in the *AP2* family (Klucher et al., 1996). Line S862 was shown to have an SNP in the linker region of the *5AQ* gene causing a Thr-to-Ile substitution and exhibited a speltoid spike morphology, indicating that this linker region of *5AQ* was important for its proper function, similar to other *AP2* family genes.

A previous study revealed that miR172 can downregulate the expression of the *Q* gene by cleaving its transcripts, and a mismatch at the miR172 target site can result in the formation of a compact spike (Sormacheva et al., 2015; Debernardi et al., 2017; Greenwood et al., 2017). Line S202 had an SNP at the miR172 target site that resulted in a dwarf plant height and compact spike, consistent with a previous study (Sormacheva et al., 2015; Debernardi et al., 2017; Greenwood et al., 2017). In addition, line S175 exhibited an SNP at the predicted MYB TF target site of exon 10 (PlantCARE). Line S175 showed a compact spike and an extremely reduced plant height, which let us to remember the regulatory effect of miR172 on the *Q* gene. In *Arabidopsis*, *AtMYB125*/*DUO1*, *AtMYB33*, *AtMYB65*, and *AtMYB38* control floral organ development (Dubos et al., 2010). In addition, the *MYB* family is involved in the cell wall regulatory network that controls cellulose and lignin biosynthesis (Rao and Dixon, 2018). Interestingly, many *MYB* family DEGs were identified from previous transcriptome data and our RNA-seq data, indicating that MYB family TFs may interact with Q (Zhang et al., 2020). Therefore, we suspected that the MYB TF is upstream of the *Q* gene.

### The Spike Morphology Variation Was Potentially Attributed to Translation Termination and Reduced Expression

We sampled young spikes from the glume primordia stage (W3) to the heading stage (W10) and used qRT-PCT to quantify the expression levels of the *Q* gene. The expression levels showed a trend of first increasing and then decreasing, consistent with previous studies in CS (Figure 6B; Simons et al., 2006). The *q* allele was expressed at lower levels than the *Q* allele throughout spike development, but herein, the *Q-e5t* allele was expressed a the same level and exhibited the same trend as the *Q* allele at the W3-W5 stages, which may have been attributed to the *Q-e5t* allele having the same miR172 target site sequence as the *Q* gene (Simons et al., 2006; Debernardi et al., 2017; Liu et al., 2018). At the W6 scale, the expression level of the *Q-e5t* allele was rapidly reduced relative to that of the *Q* allele, suggesting that its transcript was rapidly degraded by some mechanism. Combined with the phenotype data, these results revealed that the spike phenotype gradually appeared at the W5-6 scales (Figures 6A,B). Based on the above analysis, we hypothesize that the speltoid spike morphology of *je0275* was caused by a combination of protein truncations and differential expression at the W6 scale.

Several early terminated mutated alleles were reported in previous studies, but they showed differential transcription levels. The *Q^t^* allele, which had a transposon insertion in exon five and terminated early in exon seven, showed the same expression level as the *Q* allele at the young spike GS24 stage, which was consistent with our results (Jiang et al., 2019). However, the *Q-mut* allele that terminated early in the second exon was expressed at lower levels than the *Q* allele at the W5.5–9 scales, possibly due to having different mutated sites or different genetic backgrounds (Zhang et al., 2020).

Interestingly, the *Q* gene produced two other transcripts in addition to the full-length CDS via alternative splicing (Figures 7A,B). These three transcripts did not differ in the first eight exons, and all had complete *AP2* domains, indicating that their proteins may function properly. However, T1 was expressed at the highest level, followed by T3, and T2 was expressed at the lowest levels at the W6 and W9 scales (Figure 7D). The *Q* and *Q-e5t* alleles had the same alternative splicing pattern, indicating that the mutation in exon 5 of the *Q-e5t* allele may not have affected the alternative splicing that occurred at exon 9. In addition, the *Q* gene had different alternative splicing patterns in nodes, internodes and flag leaves relative to those in spikes. Among the three transcripts, T3 was expressed at the highest level, followed by T1, and T2 was expressed at the lowest level (Figure 7D). We suspected that the high expression level of T3 may have been due to the missing miR172 target site. In addition, our study and numerous others have revealed that the *Q* gene influences plant height (Kato et al., 2003; Jantasuriyarat et al., 2004; Jiang et al., 2019; Song et al., 2019). We found that the expression levels of *Q* were higher in nodes than in internodes and flag leaves, indicating the potential involvement of all transcripts or only T3 in the regulation of plant height (Figure 7E).

### The Putative Regulatory Network of Q

Downstream target genes are the bridge connecting TFs and their regulated traits. DAP-seq revealed 630 genome-wide binding sites located in genic regions, including 112 putative target genes with binding sites mapped to the proximal promoter. These data in combination with RNA-seq data identified seven genes with significant differences in expression, including six downregulated genes and one upregulated gene (Figure 8F).

TraesCS1B02G214400 (*TaSCE1*) is the homolog of *OsSCE1*, which can conjugate the small ubiquitin-like modifier (SUMO) to proteins and respond to drought stress in rice (Nurdiani et al., 2018). Another SUMO-conjugating enzyme, *SaSce9*, from *Spartina alterniflora*, can enhance salinity and drought stress tolerance in *Arabidopsis* (Karan and Subudhi, 2012). Compared with wild wheat, domesticated common wheat always loses tolerance to drought stress due to artificial environments (Budak et al., 2013). Therefore, we suspected that the *Q* gene may affect salinity and drought stress tolerance by interacting with *TaSCE1*. TraesCS7B02G488200 belongs to the pfkB-like carbohydrate kinase family protein, which includes a variety of carbohydrate kinases, such as fructokinase (Wu et al., 1991). Fructokinase-like proteins 1 and 2 (FLN1 and FLN2) were identified in *Arabidopsis* and shown to play important roles in chloroplast development (Arsova et al., 2010). The *fln* mutant showed fewer inflorescences and severe chlorotic phenotypes, indicating that the FLN protein is indispensable for plant growth and development (Gilkerson et al., 2012). TraesCSU02G263200 was annotated as a subunit 12 of mediator of RNA polymerase II (*TaMED12*), which was identified as a cyclin-dependent kinase (CDK) module in yeast (Guglielmi et al., 2004). *MED12*/*CRP* enhances *FT* and can promote flowering by regulating *SOC1*, *FUL*, *AP1*, and *FLC* in *Arabidopsis*. In addition, *crp* loss-of-function mutants showed various floral defects, indicating that *MED12* was also critical for floral development (Imura et al., 2012). Interestingly, *je0275* and varieties with previous loss-of-function mutants of the *Q* gene all flowered earlier than the WT, indicating that Q may interact with *TaMED12* to regulate *FT* and heading time (Debernardi et al., 2019). Notably, we found that *TaGRF1-2D* was bound by Q at its 1,060 bp upstream. *GRF* is a class of plant-specific TFs that play an important role in regulating leaf and stem growth, floral organ development and responses to stress (Omidbakhshfard et al., 2015). In *Arabidopsis*, *AtGRF1-9* can interact with *AP1* and *SEP3* and then regulate flower development and sepal-petal identity determination (Pajoro et al., 2014). The AP2 family TF ANT and the transcriptional adaptor protein SEU play important roles in ovule development. An overrepresented number of *GRF* genes were downregulated in the transcriptomes of *ant/seu* double mutants that failed to form ovule primordia, suggesting that *GRF* genes are involved in the regulation of floral organ development by *AP2* family genes (Wynn et al., 2011). In maize, *ZmGRF11*-*ZmGIF2* and *ZmGRF2*-*ZmGIF3* accelerated inflorescence stem growth in transgenic *Arabidopsis* and showed an expression preference for immature young ears; thus, these genes might be associated with the regulation of ear development (Zhang D.F. et al., 2008). *OsGRF10*, the close homolog of *TaGRF1-2D*, had high expression levels in young inflorescences. The *osgrf6/osgrf10* double mutant showed abnormal florets, including open husks, long sterile lemmas and missing paleas, highlighting the free-threshing characteristic and soft glumes of the *Q* allele (Liu et al., 2014). The soft glumes in domesticated common wheat were actually sterile lemmas, and the *Q* gene was shown to control the conversion from tough glumes in undomesticated *Ae. Tauschii* to soft glumes in domesticated wheat (Song et al., 2019). Therefore, we suspected that *TaGRF1-2D* plays an important role in the ability of the *Q* gene to control threshability, glume tenacity and spike morphology. Interestingly, we found that *TaMGD-6B* was the target gene of Q and was upregulated in *je0275*. Overexpression of *TaMGD-6B* significantly increased the number of grains per spike and the grain weight per plant but did not influence the 1,000 grain weight in transgenic rice lines (Du et al., 2020). As a previous study also reported that the *q* allele had higher grain numbers per spike, we speculated that the *Q* gene affects numerous yield traits by regulating *TaMGD-6B* (Xie et al., 2018).

We proposed a possible regulatory network of the *Q* gene (Figure 8G). On the one hand, Q interacts with *TaGRF1* to regulate threshability, glume tenacity and spike morphology; Q interacts with *TaMED12* to regulate *FT* and then affects flowering time; Q regulates pfkB family genes to affect chloroplast development and photosynthesis; and Q regulates *TaSCE1* to affect drought stress tolerance. On the other hand, Q interacts with *TaMGD-6B* to regulate the number of grains per spike and the grain weight per plant, thereby affecting the yield.

## Data Availability Statement

The original contributions presented in the study are publicly available. This data can be found here: All sequencing datasets for this study can be found in the National Center for Biotechnology Information (NCBI) under the BioProject ID PRJNA774162, PRJNA774156, and PRJNA774423 with the Sequence Read Archive (SRA) submission ID SUB10560636, SUB10564928, and SUB10567318, respectively.

## Author Contributions

LL conceived the project and revised the manuscript. HX designed the experiment. JZ performed most of the experiments and data analysis. HG developed the mutant. YL assisted with the protein expression. XX drew some of the pictures. YX, LZ, JG, SZ, and YD participated in field trials. All authors have read and approved the final manuscript.

## Conflict of Interest

The authors declare that the research was conducted in the absence of any commercial or financial relationships that could be construed as a potential conflict of interest.

## Publisher’s Note

All claims expressed in this article are solely those of the authors and do not necessarily represent those of their affiliated organizations, or those of the publisher, the editors and the reviewers. Any product that may be evaluated in this article, or claim that may be made by its manufacturer, is not guaranteed or endorsed by the publisher.

## Footnotes

1
http://www.wheatgenome.org/

2
http://polymarker.tgac.ac.uk/
