# Supplementary material for: Identification of the Q Gene Playing a Role in Spike Morphology Variation in Wheat Mutants and Its Regulatory Network
Source: Front Plant Sci. 2022 Jan 11;12:807731. doi: 10.3389/fpls.2021.807731 (PMC8787668; doi:10.3389/fpls.2021.807731)
Supplement: Supplementary file 1 [file Data_Sheet_1.docx]

Supplementary Material

# Supplementary Figures and Tables

## Supplementary Figures


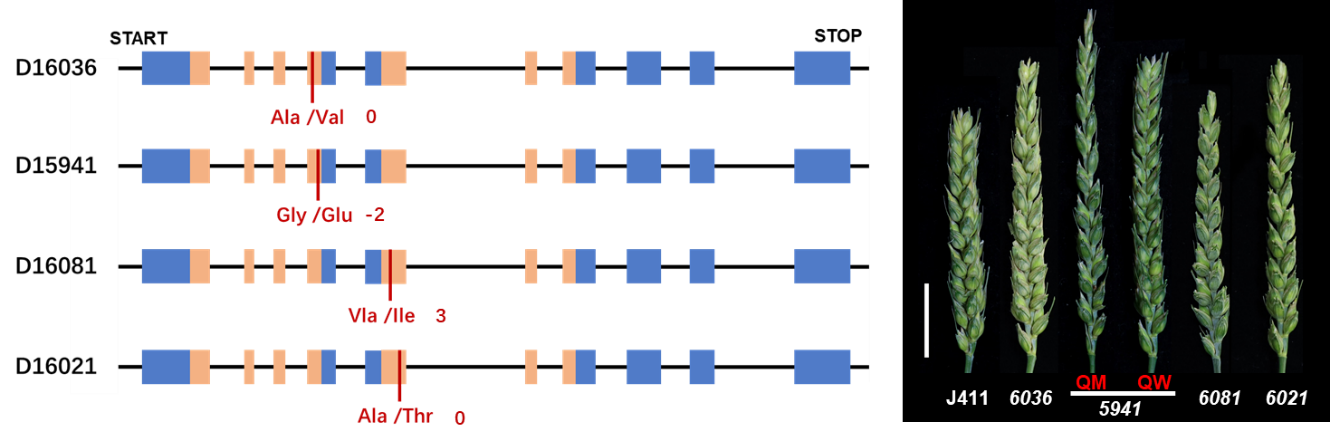
**Supplementary Figure 1.** The thousand grain weight, grain length, grain width and grain length/width of *je0275* and WT plants, grown in approximately 10 rows, were measured. The data are presented as the mean, and the error bars indicate the SD. * indicates significant differences at the 0.05 level (Student’s t test).

**Supplementary Figure 2.** Mutation sites and phenotypes of M_3_ mutant lines with mutations in the *5AQ* gene.


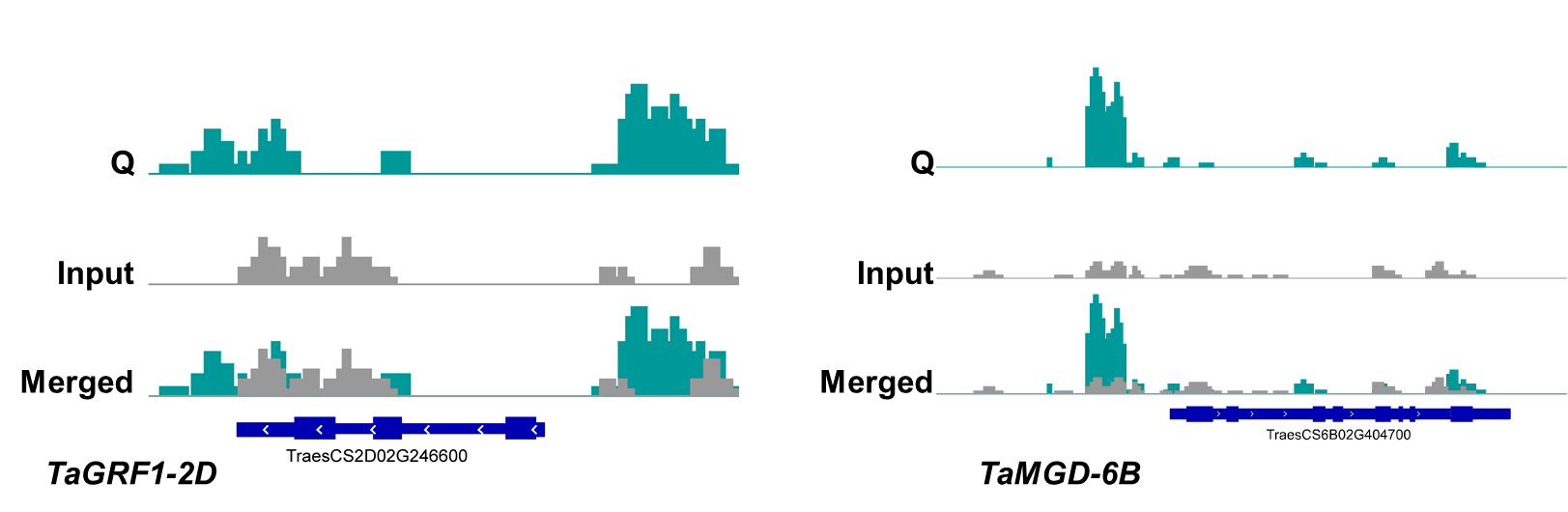

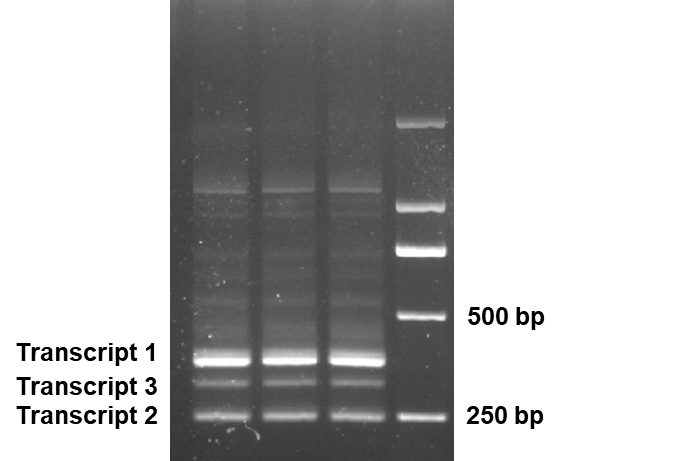
**Supplementary Figure 3.** The *Q* gene can produce three transcripts as determined by RT-PCR.


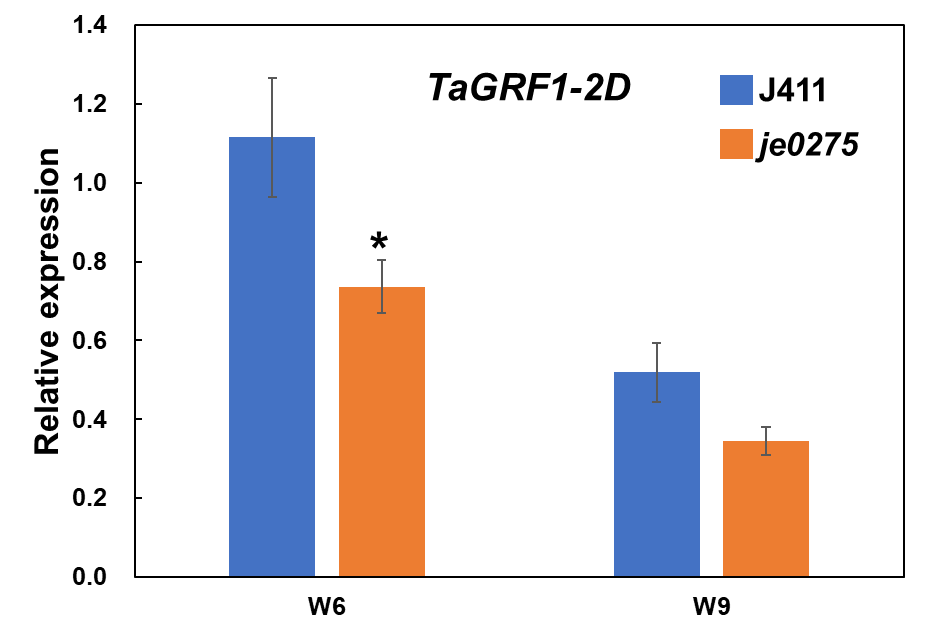
**Supplementary Figure 4.** Q can bind to the promoters of *TaGRF1-2D* (-1060 bp) and *TaMGD-6B* (-936 bp). Input was used as the negative control.

**Supplementary Figure 5.** The RNA-seq results were confirmed by qRT-PCR at the W6 and W9 scales. The data are presented as the mean, and the error bars indicate the SD. * indicates significant differences at the 0.05 level (Student’s t test).

## Supplementary Tables

**Supplementary Table 1.** Primers used in this study.

| **Primer** | **Sequence (5'-3')** | **Note** | |
| --- | --- | --- | --- |
| **KASP primers** | | |
| PQ389A | CGTCCAGCTCGGTGAACC | Mapping | |
| PQ389B | CGTCCAGCTCGGTGAACT |
| PQ389R | CGGTCGGGGAGGTCATCT |
| Q188A | GAAGCCCGGACAAGGTTACT | Mapping | |
| Q188B | GAAGCCCGGACAAGGTTACC |
| Q188R | TGATGATAGTAACACTTCTTCTAG |
| PQ205A | GTTTCGTGGTCATGTGAGATGCG | Mapping | |
| PQ205B | GTTTCGTGGTCATGTGAGATGCA |
| PQ205R | GGTGAATGCGGTTGCCCC |
| PQ309A | GTTACCTTGTCCCACAGAACTG | Mapping | |
| PQ309B | GTTACCTTGTCCCACAGAACTA |
| PQ309R | CCCTGATATAACAGAAAATGGTTGT |
| P05A | AGGACCAAAAGCATCCCTTAGG | Mapping | |
| P05B | AGGACCAAAAGCATCCCTTAGA |
| P05R | TCGGGAAGAGAAGCATGGTG |
| **For cloning full-length sequence of *5AQ*/*5Dq*** | | | |
| Q-P1-F | ACGCAACAATGGCGGACTGCTGT | Part 1 of *5AQ* | |
| Q-P1-R | ACCAAGTAGTGCGCCGGATACAT |
| Q-P2-F | TAGTGTCAAGTTCATGAGCAGTC | Part 2 of *5AQ* | |
| Q-P2-R | GGCGAGGAGTGCGTTTTATTCGGT |
| Q-D1F | CGGTGGGTCAAGCGAGTTTCGGAG | *5Dq* (Zhao et al., 2018) | |
| Q-D5R | CCGTGATCCTGATCATGGCAACGCC |
| **Diagnostic markers** | | | |
| Q-CAPS-3F | TCTGTGAAATTTCATTTCAACTG | Identification of *Q-e5t* allele | |
| Q-CAPS-3R | TTTGAAGCAGGTAATCTTATCC |
| **qRT-PCR primers** | | | |
| 5ARQ-F | GGATCTGCGGATGTCGCAACCC | Transcript 1 of *5AQ* (Zhao et al., 2018) | |
| 5ARQ-R | ATGCACAGGCCACTGGGAC |
| qT2-F | TGCTGATGCTCTTGACTTGGATCTG | Transcript 2 of *5AQ* | |
| qT2-R | GCACCTGCACCTGAGAAGAGAT |
| qT3-F | TAGAAAATTTGCTGTTGGCCCAGT | Transcript 3 of *5AQ* | |
| qT3-R | AGCGATATCAAAAGGCACAAATATC |
| GRF1-qF | GAAGGAGGCAAGAAGACTGAAA | *TaGRF1-2D* | |
| GRF1-qR | GCTAGACAACACTAAAGTCTTTC |
| Actin.F | ATGGAAGCTGCTGGAATCCAT | Wheat Actin (Zhang et al., 2011) | |
| Actin.R | CCTTGCTCATACGGTCAGCAATAC |
| **RT-PCR primers** | | | |
| QAS-F | TTCCCCTGAATCGTCAACCACAATGA | Detection of AS of *5AQ* | |
| QAS-R | AGAACCGGTGGTGGTCCGGGTACGG |
| QCDS-F | GATGGTGCTGGATCTCAATGTG | Amplification of full-length CDS of *5AQ* | |
| QCDS-R | TTCAGTTGTCCGGCGGGCGGGGGAA |

**Supplementary Table 2.** Genetic segregation analysis of mutant *je0275* in the reciprocally crossed F_2_ populations.

|  |  | **Observed** | **Expected** | **χ^2^** | ***P* (df=1)** |
| --- | --- | --- | --- | --- | --- |
| J411×*je0275* | Speltoid spike plants | 235 | 232 | 0.18 | 0.67 |
|  | Normal spike plants | 74 | 77 |  |  |
|  | Total | 309 |  |  |  |
| *je0275*×J411 | Speltoid spike plants | 146 | 150 | 0.60 | 0.44 |
|  | Normal spike plants | 54 | 50 |  |  |
|  | Total | 200 |  |  |  |

**Supplementary Table 3.** The genotype of *Q* gene in F_2_ populations.

|  | **J411 genotype** | ***je0275* genotype** | **Heterozygous** |
| --- | --- | --- | --- |
| Normal spike plants | 171 | 0 | 0 |
| Speltoid spike plants | 0 | 120 | 351 |

**Supplementary Table 4.** The mutation sites and phenotype of mutants with mutations in *5AQ*/*5Dq* genes.

| **Lines** | **CDS position** | **Predicted translational changes** | **Domain/Motif** | **Genotype** | **Spike morphology** | **Plant height (cm)** |
| --- | --- | --- | --- | --- | --- | --- |
| S334 | C-358-T | Q-120-STOP | AP2-R1 | Homozygous | Spelta | 83.60 |
| S325 | *AQ*: G-487-A *Dq*: C-620-T | *AQ*: A-163-T  *Dq*: A-207-V | *AQ*: AP2-R1  *Dq*: Linker | Homozygous | Spelta | 88.30 |
| S862 | C-572-T | T-191-I | Linker | Homozygous | Spelta | 95.00 |
| S316 | G-672-A | W-224-STOP | AP2-R2 | Homozygous | Spelta | 92.10 |
| S328 | G-672-A | W-224-STOP | AP2-R2 | Homozygous | Spelta | 91.56 |
| S847 | G-672-A | W-224-STOP | AP2-R2 | Homozygous | Spelta | 94.20 |
| S850 | G-672-A | W-224-STOP | AP2-R2 | Homozygous | Spelta | 96.00 |
| S856 | G-672-A | W-224-STOP | AP2-R2 | Homozygous | Spelta | 98.60 |
| S859 | G-672-A | W-224-STOP | AP2-R2 | Homozygous | Spelta | 100.10 |
| S175 | C-1223-T | P-408-L | MYB site (pridicted) | Homozygous | Compact | 46.09 |
| S202 | C-1238-T | A-413-V | MiR172 target site | Homozygous | Compact | 54.00 |
| D16036 | C-485-T | A-162-V | AP2-1 | Homozygous | Spelta |  |
| D15941 | G-503-A | G-168-E | AP2-1 | Heterozygous | Spelta |  |
| D16081 | G-646-A | V-216-I | AP2-2 | Homozygous | Spelta |  |
| D16021 | G-676-A | A-226-T | AP2-2 | Homozygous | Spelta |  |

**Reference**

Zhao K., Xiao J., Liu Y., Chen S., Yuan C., Cao A., et al. (2018). *Rht23* (*5Dq′*) likely encodes a *Q* homeologue with pleiotropic effects on plant height and spike compactness. *Theoretical and Applied Genetics*, 131, 1825–1834, 10.1007/s00122-018-3115-5.

Zhang, Z., Belcram, H., Gornicki, P., Charles, M., Just, J., Huneau, C., et al. (2011). Duplication and partitioning in evolution and function of homoeologous *Q* loci governing domestication characters in polyploid wheat. *Proc. Natl. Acad. Sci. U. S. A.* 108, 18737–18742. doi:10.1073/pnas.1110552108.
